# False Positive FDG PET/CT of Recurrent Testicular Tumour Due to Orchitis

**DOI:** 10.4274/Mirt.256

**Published:** 2014-02-05

**Authors:** Robert Mansberg, Bao Ho, Chuong Bui

**Affiliations:** 1 Nepean Hospital, Nuclear Medicine and PET, Penrith, Australia; 2 University Sydney, Discipline Imaging, Sydney, Australia

**Keywords:** Positron-emission tomography, 18FDG, testicular cancer, orchitis

## Abstract

A 47-year-old male with a history of right sided orchidectomy for stage 1 seminoma 6 months previously, was referred for a FDG PET-CT scan for restaging of testicular cancer having experiencing left testicular discomfort. Abnormally increased glyoclytic metabolism of the left testis and the inferior scrotal sac was demonstrated on the initial FDG PET-CT study. Subsequent ultrasound showed subtle heterogeneous echotexture with mild hypervascularity and no focal lesion was identified. The patient was subsequently treated with antibiotics for a presumed diagnosis of orchitis. A progress FDG PET-CT study 2 months later confirmed the complete resolution of the increased glycolytic metabolism in the left testis and the inferior scrotal sac.

**Conflict of interest:**None declared.

## Abstract (tr)

Altı ay önce evre 1 seminom nedeniyle sağ orşiektomi öyküsü olan 47 yaşındaki erkek hasta, sol testisinde rahatsızlık yakınması nedeniyle, testis kanserinin yeniden evrelenmesi için FDG PET-BT’ye refere edildi. İlk FDG PET-BT çalışmasında sol testis ve skrotum inferiorunda anormal artmış glikolitik metabolizma olduğu gösterildi. Daha sonra yapılan ultrasonografide hafif hipervasküler belirgin heterojen ekojenite mevcuttu ve hiçbir fokal lezyon saptanmadı. Daha sonra hasta, olası orşit tanısıyla antibiotik tedavisi aldı. İki ay sonra yapılan takip FDG PET-BT çalışması, sol testis ve skrotum inferiorunda izlenen artmış glikolitik metabolizmanın tamamen kaybolduğunu ortaya koydu.

## INTRODUCTION

Testicular cancer is a rare cancer in males that originates in the testicles. Initial diagnosis and staging utilises ultrasound and CT (1) with increasing use of PET/CT (2). There is also a growing role for PET/CT in surveillance following treatment (3). We present a 47 year old male with stage 1 seminoma who underwent a PET/CT surveillance following treatment. The scan demonstrated hypermetabolism in the left testicle and inferior scrotal sac and was suspicious for malignancy in the remaining testis. A progress scan 2 months following antibiotic treatment confirmed resolution of the hypermetabolism. This illustrates another inflammatory/infective cause of a false positive PET scan, due to orchitis, which in the setting of testicular cancer is essential to recognise.

## CASE REPORT

**Figure 1:**
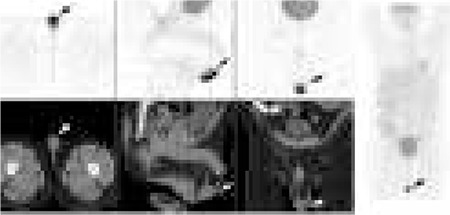
Intense FDG uptake in the left testis and the inferior left scrotal sac (arrow)

A 47 year old male with a history of right sided orchidectomy for stage 1 seminoma with left testicular discomfort was referred for a FDG PET-CT scan. FDG PET-CT was performed following administration of 5mCi (200 MBq) 18FDG on a Philips Gemini 64 Time of Flight PET/CT camera. Intense FDG uptake (SUV_max_ 19.8) was demonstrated in the left testis and the inferior left scrotal sac ((arrow) Figure 1).

**Figure 2:**
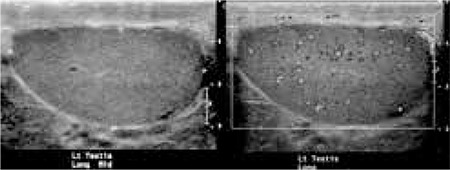
Ultrasound shows mildly heterogeneous echotexture in the left testis with mild hypervascularity and thickened scrotal skin

An ultrasound performed 2 days later demonstrated mildly heterogeneous echotexture in the left testis with mild hypervascularity and thickened scrotal skin. No focal testicular mass lesion was detected (Figure 2).

**Figure 3:**
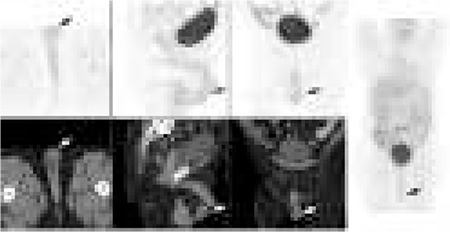
Complete resolution of the previously increased FDG avid left testis and the left inferior scrotal sac (arrow)

In the absence of a structural lesion on ultrasound examination, a presumptive diagnosis of orchitis was made and the patient was treated with a course of antibiotics with resolution of symptoms. Two months later the patient underwent a repeat FDG PET–CT study on the Philips Gemini Time of Flight 64 PET/CT camera which revealed complete resolution of the previously increased FDG avid left testis and the left inferior scrotal sac (arrow) (Figure 3).

## LITERATURE REVIEW AND DISCUSSION

Testicular cancer is a rare tumour, representing about 1% in all cancers in males, subdivided into seminomatous and nonseminomatous groups. Diagnostic procedures include physical examination, serum tumour marker (for nonseminomatous tumours) and imaging. Scrotal ultrasound can deliniate the intrascrotal mass while CT scanning is usually employed for staging (1).

The normal testis demonstrates variable FDG uptake. There may be a moderate correlation between decreasing testicular FDG uptake and increasing age, probably as a result of age-related decline in androgen production. The diagnostic utility of PET in the assessment of testicular cancer is increasingly recognized. Seminomatous tumours are in general more FDG avid than the nonseminomatous counterpart and the pattern of FDG uptake is often heterogeneous (2,3). Thus far, for staging, PET has been proven to be more sensitive and more specific than CT modality and serum tumour markers. In addition, FDG PET not only detects retroperitoneal relapse earlier than CT, it also has an advantage over CT in restaging with a high negative predictive value in predicting treatment-related fibrosis (2,3,4).

However, there are pitfalls with FDG PET where false positive results could occasionally be seen in infective/inflammatory processes as well as reactive inflammatory response post chemotherapy (5,6,7,8,9).

In this case, further ultrasonographic correlation to exclude underlying anatomical lesion was particularly helpful. The presumptive diagnosis of orchitis was confirmed by resolution of abnormal FDG uptake on progress imaging.
